# Unveiling the genomes and secondary metabolomes of *Streptomyces* spp. from freshwater sediments

**DOI:** 10.3389/fmicb.2026.1793713

**Published:** 2026-04-20

**Authors:** Inmaculada Tocino-Márquez, Martin Zehl, Jovana Batajic, Joana Séneca, Petra Pjevac, José Murillo-Alba, Jesús Martín, Olga N. Sekurova, Sergey B. Zotchev

**Affiliations:** 1 Division of Pharmacognosy, Department of Pharmaceutical Sciences, University of Vienna, Vienna, Austria; 2 Doctoral School in Microbiology and Environmental Science, University of Vienna, Vienna, Austria; 3 Department of Analytical Chemistry, Faculty of Chemistry, University of Vienna, Vienna, Austria; 4 Institute of Science and Technology Austria, Klosterneuburg, Austria; 5 Centre for Microbiology and Environmental Systems Science, University of Vienna, Vienna, Austria; 6 Joint Microbiome Facility of the Medical University of Vienna and the University of Vienna, Vienna, Austria; 7 Fundación MEDINA, Armilla, Granada, Spain

**Keywords:** biosynthetic gene clusters, fresh-water sediment, genomes, secondary metabolites, *Streptomyces* bacteria

## Abstract

Several *Streptomyces* strains were isolated from freshwater sediments collected in the Laxenburg ponds (Lower Austria). Genome sequencing and bioinformatics analyses revealed biosynthetic gene clusters (BGCs) that may specify production of chemically diverse secondary metabolites. Various culture conditions were employed to induce metabolite production, and subsequent LC-MS analyses facilitated the identification of the produced compounds and their correlation with the corresponding BGCs. These analyses of sediment-derived *Streptomyces* spp. highlight their extensive biosynthetic potential, revealing a diverse range of bioactive secondary metabolites, including siderophores, antibiotics, and other compounds with potential therapeutic applications. Genomes of two *Streptomyces* isolates, one of them representing a potentially new species, harbored several uncharacterized BGCs that may specify biosynthesis of novel secondary metabolites. Although targeted overexpression of pathway-specific regulators from these BGCs did not yield additional metabolites, whereas knockout experiments led to metabolic changes, presumably reflecting regulatory or compensatory interactions between multiple biosynthetic pathways. Continued exploration of these strains and their BGCs may lead to the discovery of new bioactive molecules with pharmaceutical and biotechnological applications.

## Introduction

*Streptomyces* is a genus of Gram-positive bacteria of the phylum Actinomycetota (previously known as Actinobacteria) renowned for its extensive capacity to produce secondary metabolites. Members of this bacterial phylum are responsible for generating more than 70% of all known antibiotics and for synthesizing a diverse array of pharmacologically valuable compounds, including antitumor and immunosuppressive agents (Harir et al., 2018; Das et al., 2023). Secondary metabolites are typically produced by *Streptomyces* during the transition from vegetative growth to the formation of aerial mycelium, a developmental stage associated with nutrient limitation and intense microbial competition (Seipke et al., 2012). Such ecological pressures are thought to have driven the evolution of complex biosynthetic capabilities in actinomycetes inhabiting densely populated environments such as soils and sediments.

Genomic analyses have revealed that *Streptomyces* species possess large genomes that encode an extensive repertoire of biosynthetic gene clusters (BGCs), typically ranging from 20 to 40 per genome (Baltz, 2017). These clusters specify the biosynthesis of chemically diverse natural products, including polyketides, non-ribosomal peptides, ribosomally synthesized and post-translationally modified peptides (RiPPs), and terpenoids. Recent advances in bioinformatics tools, such as antiSMASH (Blin et al., 2023) have highlighted the remarkable biosynthetic potential of *Streptomyces*, revealing that many BGCs show little similarity to previously characterized pathways and may therefore encode undiscovered natural products (Mohite et al., 2025). However, a major challenge in natural product research remains the functional characterization of these predicted clusters, as many of them are poorly expressed or completely silent under standard laboratory conditions.

Because bioinformatics-based genome analysis alone cannot determine whether a predicted BGC is expressed or what metabolite it produces, integrative strategies combining genomics with metabolomics have become increasingly important for natural product discovery. High-resolution mass spectrometry-based metabolomics allows the detection and characterization of metabolites produced by a microorganism, while bioinformatics-guided genome mining predicts its biosynthetic capacity. Correlating metabolomic profiles with predicted biosynthetic pathways enables the assignment of metabolites to their corresponding BGCs and helps to prioritize clusters that may encode novel compounds. Such genome-to-metabolome correlation approaches have significantly accelerated the discovery and annotation of microbial natural products in recent years (Kersten et al., 2013; Duncan et al., 2015; Wang et al., 2016; Ziemert et al., 2016). Several discovery pipelines have been developed to establish links between metabolites and their cognate BGCs. For example, combination of tandem mass spectrometry data with genomic predictions of glycosylation enzymes was used to identify glycosylated natural products and their biosynthetic origins (Kersten et al., 2013). Similarly, molecular networking approaches enabled the organization of MS/MS data into families of structurally related metabolites, facilitating dereplication and discovery of new compounds in complex extracts (Wang et al., 2016). While these approaches are powerful for identifying metabolite families and predicting biosynthetic relationships, the inferred links between metabolites and BGCs require experimental validation. Genetic manipulation provides an important complementary strategy for confirming the function of candidate BGCs and for activating cryptic metabolic pathways. Approaches such as targeted gene inactivation, overexpression of pathway-specific regulators, and heterologous expression in engineered host strains have been widely used to establish cluster-metabolite relationships and to uncover previously undetected secondary metabolites (Rutledge and Challis, 2015; Pham et al., 2021). In *Streptomyces*, engineering of BGC regulation has proven effective in modulating the expression of biosynthetic pathways, although the outcomes are often influenced by complex regulatory interactions among multiple metabolic networks.

Aquatic environments, including freshwater sediments, represent a rich reservoir of actinomycetes with significant biosynthetic potential. Nutrient-rich detrital material present in such habitats provides ecological niches that support diverse microbial communities capable of producing a wide range of secondary metabolites (Eccleston et al., 2008; Ngamcharungchit et al., 2023). Previous studies have demonstrated the value of freshwater sediments as sources of bioactive actinomycetes. For example, investigations of actinomycetes isolated from the Tagus River estuary resulted in the identification of numerous strains, including the proposed new species *Streptomyces meridianus*, which produced several antimicrobial compounds active against clinically relevant pathogens (Dos Santos et al., 2024). Zothanpuia et al. (2018) reported the isolation of diverse actinomycetes from freshwater sediments that produced antimicrobial metabolites active against Gram-positive and Gram-negative bacteria as well as yeasts. Additional studies have demonstrated that aquatic *Streptomyces* isolates can produce structurally novel compounds with promising biological activities, such as the alpiniamide polyketides isolated from *Streptomyces* sp. QHA48 (Tang et al., 2023).

In this study, we investigated the biosynthetic potential of *Streptomyces* strains isolated from freshwater sediments collected in the Laxenburg ponds in Lower Austria. Whole-genome sequencing and bioinformatic analyses were used to identify BGCs within the genomes of the isolates. To evaluate the functional expression of these clusters, LC-MS-based metabolite profiling was used under multiple cultivation and extraction conditions. Selected isolates were subjected to targeted genetic manipulation, including overexpression of pathway-specific regulatory genes and gene knockout experiments, in order to probe the functional roles and regulatory interactions of candidate biosynthetic pathways. By integrating genome mining, metabolomic analysis, and genetic manipulation, this study provides new insights into the secondary metabolite biosynthetic capacity of freshwater-derived *Streptomyces* and highlights the potential of these environmental isolates as sources of bioactive natural products.

## Results and discussion

### Isolation and genome-based taxonomic identification of sediment-derived *Streptomyces* bacteria

**FIGURE 1:**
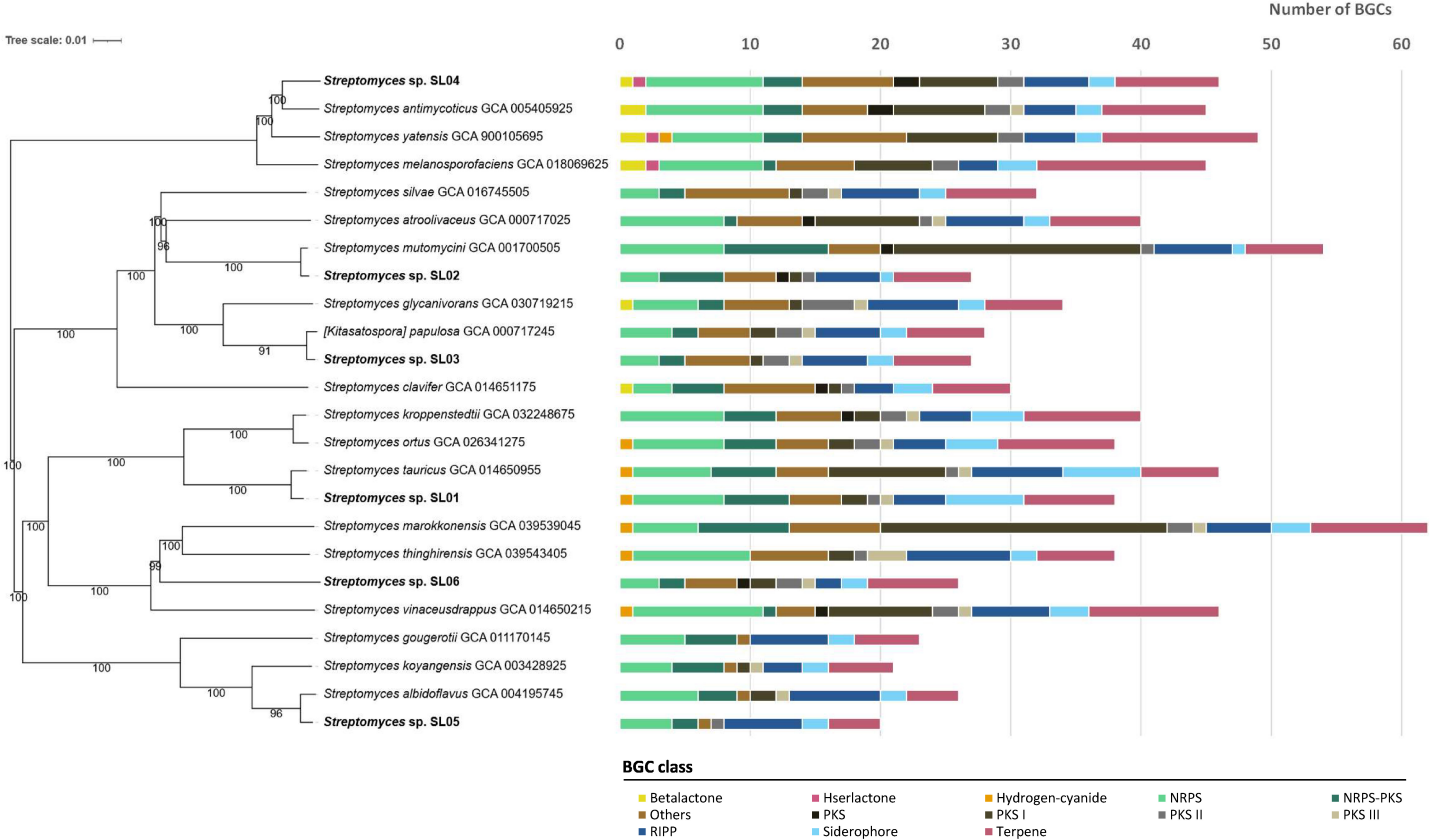
Genome-based phylogenetic trees and biosynthetic gene clusters (BGCs) detected in corresponding genomes.

Sediment samples were collected from the Laxenburg ponds in Lower Austria with the aim to specifically isolate actinomycete bacteria (see Materials and methods). This study is focused on six distinct isolates belonging to the genus *Streptomyces*, identified based on their 16S rRNA gene sequence and typical morphology. The *Streptomyces* strains were selected based on distinct colony morphology and metabolisms, such as spore formation and pigment production. Pure cultures of these isolates were obtained after several rounds of sub-culturing and subjected to whole-genome sequencing. The genome sizes of the isolates ranged from 7.19 to 10.92 Mb, with G + C content between 70.4 and 73.3% (Table 1). The assembled isolate genomes were used to generate a genome-based phylogenetic tree (Figure 1). Next, we performed the comparison of the Average nucleotide identity (ANI) and digital DNA–DNA hybridization (dDDH) values of the studied strains with reference strains closely related to them according to the inferred phylogeny (Table 2). Since strains with a dDDH ≥ 70% and ANI ≥ 95% are considered to belong to the same species, isolates SL02, SL04, and SL05 were assigned to *Streptomyces mutomycini*, *Streptomyces antimycoticus*, and *Streptomyces albidoflavus*, respectively. *Streptomyces* spp. SL01, SL03, and SL06 showed values below the established species delineation thresholds, suggesting that they may represent potential new species.

**TABLE 1:** Genomic data from six *Streptomyces* spp. from freshwater sediments.

Strain ID	Taxonomy	Genome size (Mb)	G-C content (%)	No. BGCs
SL01	*Streptomyces tauricus*	7.58	71.1%	38
SL02	*Streptomyces mutomycini*	10.58	70.7%	27
SL03	*Streptomyces papulosus*	7.68	70.4%	27
SL04	*Streptomyces antimycoticus*	7.49	71.9%	46
SL05	*Streptomyces albidoflavus*	7.19	73.3%	20
SL06	*Streptomyces* sp.	10.92	70.8%	26

Initial taxonomic classification as per genome-based phylogenetic tree (Figure 1).

**TABLE 2:** Average nucleotide identity (ANI) and digital DNA–DNA hybridization (dDDH) values between the studied strains and closely related reference strains.

SL strain genome	Reference genome	ANI (%)	dDDH	G + C difference
SL01	*S. kroppenstedtii* (GCA_032248675)	91.12	43.5	0.15
SL01	*S. ortus* (GCA_026341275)	91.12	43.5	0.13
SL01	*S. tauricus* (GCA_014650955)	91.12	91	0.13
SL02	*S. silvae* (GCA_016745505)	89.65	38.9	0.99
SL02	*S. atroolivaceus* (GCA_000717025)	89.99	39.8	0.29
**SL02**	***S. mutomycini* (GCA_001700505)**	**99.44**	**95.8**	**0.2**
SL03	*S. glycanivorans* (GCA_030719215)	93.52	53.4	0.51
SL03	*[Kitasatospora] papulosa* (GCA_000717245)	88.65	36.6	0.32
SL03	*S. clavifer* (GCA_014651175)	86.05	31.2	0.15
**SL04**	***S. antimycoticus* (GCA _005405925)**	**97.25**	**75.3**	**0.1**
SL04	*S. yatensis* (GCA_900105695)	96.20	67.7	0.18
SL04	*S. melanosporofaciens* (GCA_018069625)	95.10	60.9	0.57
SL05	*S. gougerotii* (GCA_011170145)	90.88	41.8	0.25
SL05	*S. koyangensis* (GCA_003428925)	97.75	64.5	0.3
**SL05**	***S. albidoflavus* (CGA_004195745)**	**99.05**	**91.7**	**0.09**
SL06	*S. marokkonensis* (GCA_039539045)	77.85	21.7	1.38
SL06	*S. thinghirensis* (GCA_039543405)	89.25	38.2	0.43
SL06	*S. vinaceusdrappus* (GCA_014650215)	87.91	34.4	0.54

Isolates in bold represent known species indicated in the column reference genomes.

### Secondary metabolite biosynthesis potential of *Streptomyces* isolates from sediment samples

The potential for secondary metabolite biosynthesis in the six selected *Streptomyces* spp. was assessed using antiSMASH version 7.0 software (Blin et al., 2023). The antiSMASH analysis identified a total of 184 BGCs in these genomes, represented by a variety of cluster types. The genomes were found to predominantly contain BGCs for terpenes, non-ribosomal peptide synthetases (NRPS), and ribosomally synthesized and post-translationally modified peptides (RiPPs). The *Streptomyces antimycoticus* SL04 genome contains the highest number of BGCs, with 46 clusters identified, followed by *Streptomyces tauricus* SL01 with 38 BGCs. The smallest number of 20 BGCs was detected in *Streptomyces albidoflavus* SL05. To identify BGCs that may convey the capability to biosynthesize novel compounds, of compounds that have not been reported yet, a strict threshold of < 60% similarity to known BGCs was applied. Following this standard, 76 BGCs, representing 41% of the total encoded BGCs, were found to share significant similarity with previously characterized clusters. In contrast, 108 BGCs showed no significant match to any known clusters when compared against the MiBIG database.^1^ Additional details regarding the biosynthetic potential of these six actinomycete isolates, including the identified BGCs, are provided in Supplementary Table S4.

### Identification of secondary metabolites with high-resolution LC-MS analysis of methanolic extracts

To test antimicrobial activity of the isolates, they were cultivated in three liquid media and methanolic extracts from the freeze-dried cultures tested for activity against five microorganisms. Extracts from five *Streptomyces* strains exhibited bioactivity against *Micrococcus luteus*, *Bacillus subtilis*, *Staphylococcus carnosus*, *Escherichia coli*, and *Saccharomyces cerevisiae*. These microorganisms were chosen since they represent Gram-positive and Gram-negative bacteria and yeast. No antimicrobial activity was detected in the extracts from *Streptomyces* sp. SL03 under the tested conditions (Table 3).

**TABLE 3:** Bioactivity results for extracts from strains cultured in three different media (SM17, Gause, and ISP5) are provided, with the diameter of the inhibition zone indicated in millimeters (mm).

	Inhibition zone (mm)					
SL strain ID	*M. luteus*	*B. subtilis*	*S. carnosus*	*P. putida*	*E. coli*	*S. cerevisiae*
SL04	25_*SM17*_ 21_*Gause*_ 17_*ISP5*_	14_*SM17*_ 21_*Gause*_ 15_*ISP5*_	26_*SM17*_ 26_*Gause*_ 14_*ISP5*_	–	–	17_*ISP5*_
SL03	–		–	–	–	–
SL06	12_*SM17*_	30_*ISP5*_	11_*SM17*_	–	21_*Gause*_	–
SL05	–	–	–	–	15_*Gause*_	34_*SM17*_ 31_*Gause*_
SL01	16_*ISP5*_	10_*ISP5*_	13_*ISP5*_ 10_*Gause*_	–	–	13_*ISP5*_
SL02	22_*SM17*_ 21_*Gause*_ 27_*ISP5*_	15_*ISP5*_	13_*SM17*_	–	–	16_*ISP5*_ 17_*Gause*_

Next, all methanolic extracts were analyzed by an in-house high-resolution LC-MS workflow comprising measurements on a timsTOF fleX system and non-automated data analysis utilizing GNPS, The Natural Products Atlas, and CAS SciFinder (American Chemical Society) as databases (Wang et al., 2016; van Santen et al., 2021; Vignolle et al., 2025).

In *Streptomyces* sp. SL06 extract, megalochelin (BGC 2.24) in cyclic and linear form was confirmed by thorough interpretation of the fragment ion pattern. *N*-(2,3-dihydroxybenzoyl) serine (BGC 2.21) was tentatively identified as enterobactin-related metabolite. In addition to the Micro4All Molecules Gateway workflow, we found several prenylindole-derivatives, including a 5- or 6-dimethylallylindole-3-acetaldoxime and 5- or 6-prenyltryptophol (Ozaki et al., 2013). The BGCs specifying the biosynthesis of this family of L-tryptophan metabolites are frequently found in actinomycetes and well-studied. In the corresponding BGC 2.23 in *Streptomyces* sp. SL06, the presence of a flavin-dependent monooxygenase gene next to the one encoding the tryptophan dimethylallyltransferase family protein suggests it to be a Type B gene cluster according to the classification by Ozaki et al. (2013). We also detected a large number of germicidins, including a presumably novel congener. These 4-hydroxy-2-pyrones, some of which were shown to act as autoregulatory inhibitors of spore germination, are biosynthesized by the type III PKS germicidin synthase found in BGC 2.20 (Petersen et al., 1993; Song et al., 2006; Chemler et al., 2012). The tentative identification of ribocitrin, a dextransucrase inhibitor, and PDE-I, a cyclic adenosine-3’,5’-monophosphate phosphodiesterase inhibitor, is based exclusively on the LC-MS data, since their biosynthesis is yet to be established (Enomoto et al., 1978; Takashio et al., 1982). Finally, in SM17, an abundant homolog of the ribosomal peptide PepX was assigned by *de novo* sequencing (Sekurova et al., 2022). The exact biological role of the PepX homologs, which we frequently identify in *Streptomyces* sp. cultures, is unknown.

Hydroxamate-type siderophores (BGC 1.14) were confirmed as main secondary metabolites produced by *Streptomyces albidoflavus* SL05 in SM17, with desferrioxamine E, desferrioxamine X_7_, desferrioxamine B, and a new congener with the sum formula C_31_H_52_N_6_O_10_ being the most abundant ones (Mike et al., 2017). Also, in agreement with the Micro4All Molecules Gateway results, we identified surugamide A (BGC 1.12), several polycyclic tetramate macrolactams (BGC 1.2), and several antimycins (BGC 1.20). In the case of the latter two, there are several highly similar isomers described in actinomycetes that cannot be safely differentiated based solely on LC-MS data.

In *Streptomyces tauricus* SL01, we could confirm desferrioxamines (BGC 2.16) and undecylprodigiosin congeners (BGC 2.5). The non-automated data analysis also lead to the identification of glycosylated anthracyclines, likely cinerubin B and related congeners associated with BGC 2.14 (Kersten et al., 2013). The LC-MS data of another group of compounds matched to pamamycins, macrodiolide antibiotics that also stimulate the formation of aerial mycelia, but the corresponding BGC could not be clearly identified in SL01 (Rebets et al., 2015). Finally, a group of potentially new secondary metabolites, likely NRPS products, was found.

Congeners of niphimycin (BGC 3.5), grisorixin (BGC 3.6), and desferrioxamine E (BGC 3.22) were confirmed as most abundant secondary metabolites in the cultures of *Streptomyces antimycoticus* SL04.

Venturicidin A (BGC 2.4) was tentatively identified as main component in all three culture extracts of *Streptomyces* sp. SL02. This glycosylated macrolide shows inhibitory activity toward fungal F_0_F_1_-ATPase (Rhodes et al., 1961; Li et al., 2021). Inthomycin A or phthoxazolin A (BGC 2.3), both are oxazole-containing hybrid PKS/NRPS antibiotics, and several of its congeners were also produced in all three media, but in lower amounts in Gause (Hou et al., 2020). Finally, a potentially new natural product with the sum formula C_15_H_27_NO_2_ was observed.

*Streptomyces* sp. SL03 was found to have a secondary metabolite profile very similar but not identical to *Streptomyces* sp. SL203 previously isolated from the fresh-water sponge *Spongilla lacustris* with coelichelin (BGC 1.2), detoxin S_1_ (BGC 4.6), coprisidin A and B (BGC 3.2), sceliphrolactam (BGC 1.7), and coproporphyrin detected in both strains (Graffius et al., 2023). In addition to the natural products shared with *Streptomyces* sp. SL203, deoxyguanidinoproclavaminic acid (BGC1.5), schizokinen (BGC1.9), and polycyclic tetramate macrolactams (BGC1.1) were tentatively identified in SL03 (Shrestha et al., 2017).

### Metabolite profiling of ethanolic extracts via the Micro4All Molecules Gateway

**FIGURE 2:**
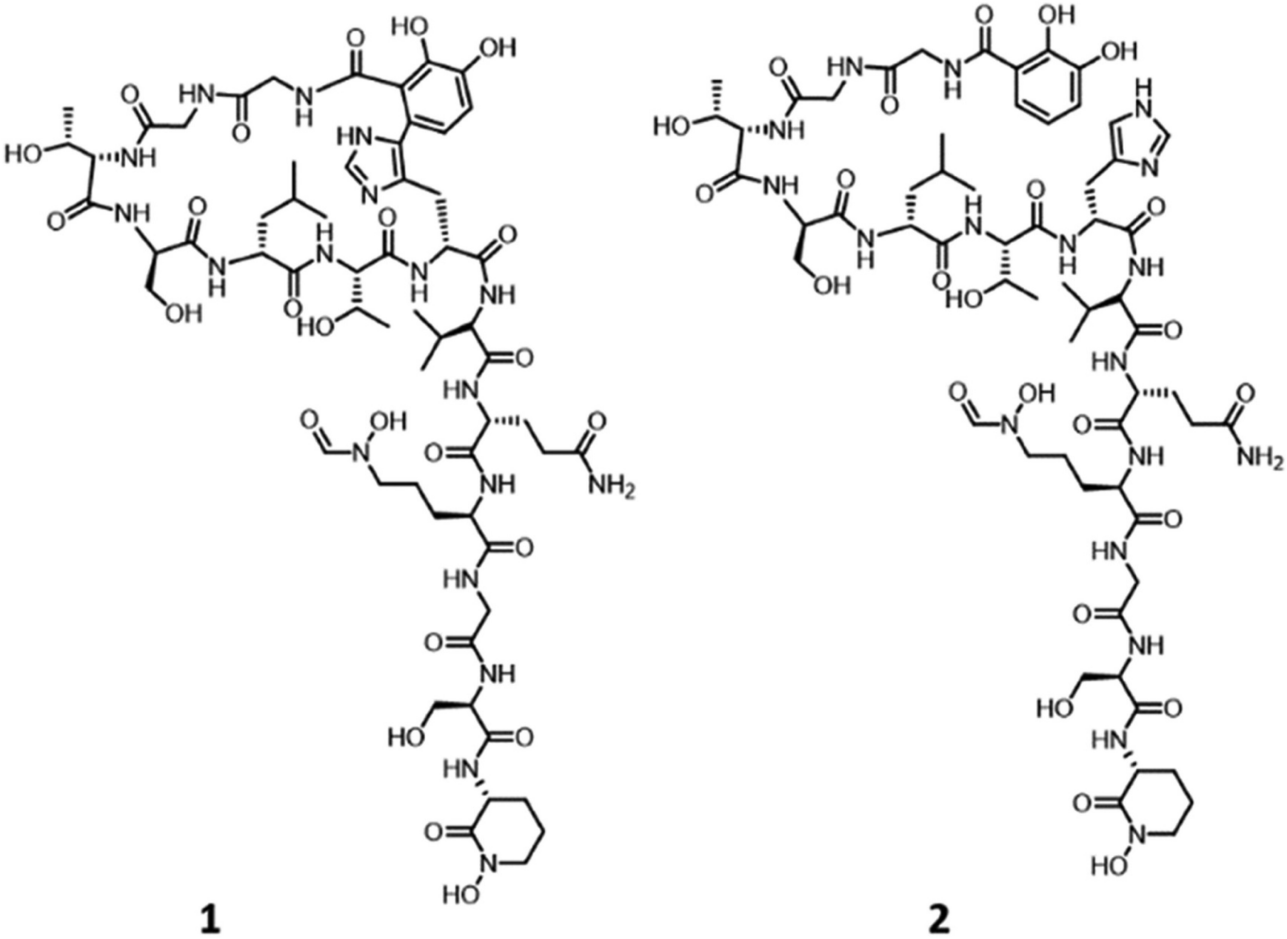
Chemical structure of cyclized (1) and linear form (2) of megalochelin.

Tentative identification of secondary metabolites was performed on extracts from the same cultures but prepared using an alternative method that included ethanol extraction and direct drying of the supernatant in microplates to ensure consistency across samples (see Materials and methods). Analysis was carried out using high-resolution LC-MS analysis on an Orbitrap Exploris 120 system (Thermo Fisher Scientific GmbH, Germany) followed by data analysis using the Micro4All Molecules Gateway (Naicons), an automated pipeline designed for high-throughput metabolomic profiling (Simone et al., 2024). Only those secondary metabolites which we were able to associate with particular BGC were taken into account, as presented in Table S6. According to this analysis, *Streptomyces* sp. SL06 produced megalochelin (BGC2.24) in media SM17 and ISP5, a siderophore involved in microbial iron acquisition (Bohac et al., 2017; Vind et al., 2023). Megalochelin (Figure 2) is the largest known and first characterized “ring-and-tail” siderophore, where the hexapeptide tail is responsible for iron binding, while the function of the cyclic heptapeptide ring remains unclear. The rigidity of the ring structure may facilitate receptor binding and modulate bioactivity, resembling acinetobactin, which enhances growth-inhibitory properties (Bohac et al., 2017). In all three media tested, SL06 produced nigericin-like compounds (tentatively associated with BGC2.2), while bonactin and enterobactin-like compounds were only produced in SM17 (associated to BGC2.21 and presumably BGC2.2). Nigericin, a polyether ionophore originally isolated from *Streptomyces hygroscopicus*, exhibits potent antibacterial effects against *Staphylococcus aureus*, *Bacillus cereus*, and *Enterococcus faecalis*, as well as several mycobacterial species (Gao et al., 2021; Zhu et al., 2022). Enterobactin-like siderophores serve a similar ecological function as megalochelin by promoting survival in iron-limited environments (Raymond et al., 2003). Bonactin, a compound originally isolated from *Streptomyces* spp. from aquatic sediment samples, has demonstrated antimicrobial activity against Gram-positive and Gram-negative bacteria as well as antifungal activity (Schumacher et al., 2003; Thiyagarajamoorthy et al., 2018). Production of non-actin and bonactin may explain the antimicrobial activity of the SM17-derived extract against 2 out of the 3 Gram-positive bacteria tested (Table 1).

According to the Micro4All platform analysis, *Streptomyces albidoflavus* SL05 produced various deferoxamines (BGC1.14) in medium SM17. Desferroxamines are iron-chelating siderophores that have therapeutic applications in iron overload disorders, and some have also antimicrobial activity (Miethke and Marahiel, 2007; Parker et al., 2023). Nocardamin, (desferrioxamine E), also identified in the bacterial extracts of this strain, is a cyclic trihydroxamate siderophore with high-affinity iron-chelating properties. By sequestering iron from competing organisms, it enhances microbial survival and competitiveness, contributing to its ability to inhibit competing organisms (Kalinovskaya et al., 2011; Lopez et al., 2019; Mahmud et al., 2022). Additional metabolites detected in the extracts of SL05 cultivated in Gause and/or SM17 medium included maltophilin, ikarugamycin epoxide and alteramides (all likely to be specified by BGC1.2), and levorin (BGC1.19). Maltophilin and ikarugamycin epoxide exhibit a diverse range of biological activities, including antimicrobial and enzyme inhibition (Jakobi et al., 1996; Bertasso et al., 2003; Elkin et al., 2016; Waack et al., 2017; Zhang et al., 2023). Previous studies have shown that alteramide B, a polyclyclic tetramate macrolactam antifungal metabolite produced by *Lysobacter* sp., exhibits potent inhibitory activity against yeast, particularly *Candida albicans* (Ding et al., 2016; Tang et al., 2021). Both media also promoted the production of antimycins (BGC1.20), a well-studied family of antibiotics with broad-spectrum activity against fungi and bacteria (Ogita et al., 2009). Additionally, surugamide A, a cyclic peptide antibiotic with broad-spectrum activity, including cytotoxic properties, was identified. This compound was initially discovered in a marine-derived strain of *S. albidoflavus*, clearly related to *S. albidoflavus* SL05 (BGC1.12) (Matsuda et al., 2019; Maw et al., 2024).

*Streptomyces tauricus* SL01 was found to produce deferoxamines (BGC2.16), nocardamin (BGC2.16), undecylprodigiosin (BGC2.5) and antimycin A17, A19 (BGC2.1) in medium SM17, with streptorubin B (BGC2.5) being produced in both SM17 and Gause. Undecylprodigiosin, a red-pigmented tripyrrole antibiotic, has been linked to antimicrobial, immunosuppressive, and cytotoxic activities (Feitelson et al., 1985; Ramesh et al., 2021; Lee et al., 2024). Similarly, streptorubin B, is a related prodiginine antibiotic metabolite produced by several *Streptomyces* species, including the model organism *Streptomyces coelicolor* (Haynes et al., 2010; Withall et al., 2015; Marshall et al., 2020).

Niphimycin and its derivative niphimycin D (BGC3.5) were produced by *Streptomyces antimycoticus* SL04 and detected in the extracts from cultures grown in all media tested. Niphimycin is an antifungal agent acting by disrupting fungal plasma membrane (Song et al., 2019; Wang et al., 2023). Other compounds detected included grisorixin, which was present in SM17- and Gause-grown cultures, and scopafungin, an antimicrobial agent which was produced in SM17 and ISP5. Both of these compounds could be associated with BGC3.5. Grisorixin is a highly toxic ionophorous antifungal and antibacterial belonging to the nigericin group (Trejo-Estrada et al., 1998; Li and Velkov, 2019; Nair and Abraham, 2020). Several metabolites were exclusively detected in SM17, including nocardamine (BGC3.22), nigericin (BGC3.6) and coelichelin (BGC3.3), a known siderophore (Challis and Ravel, 2000). Nigericin exhibits potent antibacterial activity, particularly against Gram-positive bacteria (Gao et al., 2021; Zhu et al., 2022). 23-O-butyrylbafilomycin D, a macrolide antibiotic from the bafilomycin family, is produced by actinomycetes and broadens the family’s pharmacological profile, including cytotoxic and antifungal effects (Zhang et al., 2017).

The only metabolite putatively identified in *Streptomyces mutomycini* SL02 culture extracts with Micro4All platform, which could be connected to a BGC was schizokinen (presumably BGC2.11) produced in SM17 medium. Schizokinen, like other siderophores, plays a crucial role in microbial iron acquisition and competitive survival (Mullis et al., 1971; Chuljerm et al., 2019).

*Streptomyces* sp. SL03 produced coelichelin (BGC1.2), ikarugamycin epoxide, and schizokinen (BGC1.9) in medium SM17, while both SM17 and Gause induced the biosynthesis of alteramide A and maltophilin (BGC1.1). This strain also produced hopanoids in ISP5 and Gause media (BGC1.6).

The results obtained from the ethanolic and methanolic extraction methods and using either an automated (Micro4All Molecules Gateway) or in-house manual data analysis platform, respectively, were largely confirmatory, but also complemented each other nicely to provide an overall better view of the secondary metabolomes of the *Streptomyces* isolates. Methanol is somewhat more polar than ethanol, which results in different solubilities for some metabolites. However, we assume that this mainly affects the relative composition but has little impact on the qualitative analysis, since LC-MS is a very sensitive technique with a wide dynamic range. In our opinion, the two main reasons why some compounds were uniquely detected by one method but not the other are: (1) the different databases and data sources used in the two workflows, and (2) the limitations in the number of compounds that can be dereplicated by manual data analysis due to time-constraints. Only very few compounds identified by the automated workflow from the ethanolic extracts were not found upon manual data analysis, for example bonactin in *Streptomyces* sp. SL06, levorin congeners in *Streptomyces* sp. SL05, as well as several low abundant congeners of identified secondary metabolites that were ignored in the manual workflow. On the other hand, several natural products, including highly abundant ones, were missed by the automated workflow, particularly in *Streptomyces* sp. SL06. Among the most abundant metabolites detected in this strain were germicidins A, B, C, I, and J, well-characterized autoregulatory antibiotics capable of inhibiting spore germination in *Streptomyces* and other taxa (Aoki et al., 2011). These compounds, active at picomolar concentrations, underscore the potential of SL06 to modulate microbial development (Petersen et al., 1993). Furthermore, the dimethylallylindole derivatives and ribocitrin detected by the manual workflow only might also facilitate advantages in ecological competition (Ohnuki et al., 1981). For the extracts of *Streptomyces* sp. SL03, the manual workflow also yielded products from a much larger number of BGCs, including deoxyguanidinoproclavaminic acid, detoxin S_1_, and coprisidins. In summary, while the automated (Micro4All Molecules Gateway) workflow is much faster, it yields many false positive or questionable hits that must be manually filtered out, and it misses several interesting secondary metabolites, particularly if they are described in the literature but are not yet (correctly) entered into the most relevant natural product databases.

When comparing the three different culture media used, it was obvious that for nearly all strains SM17 was favorable regarding both, the number of detected natural products and their concentrations. One obvious reason was that this medium was the only one that was not supplemented with Fe^2+^ salts and hence the only one that lead to the production of significant amounts of siderophores. In addition, this medium is the most complex one and already contains various primary and secondary metabolites, such as isoflavonoids and saponins from soy flour. These plant-derived compounds might act as elicitors on the cultured *Streptomyces* species.

### Genome mining of *Streptomyces* spp. SL03 and SL06 isolates

Since several of the BGCs identified in the isolates SL03 and SL06 appeared to be so far uncharacterized and may potentially specify production of novel bioactive compounds, these isolates were selected for genome mining. Particular focus was placed on a non-ribosomal peptide synthetase-polyketide synthase (NRPS-PKS) hybrid BGC identified in SL03 (BGC region 1.1, Supplementary Tables S5, S6) and two distinct type I PKS clusters identified in SL06 (BGC regions 2.2 and 2.19, Supplementary Tables S5, S6), none of which showed homology to previously characterized BGCs.

Given preliminary computational analysis and the bioactivity data from the strains, we selected SL03, which did not exhibit antimicrobial activity against the tested pathogens, for targeted overexpression of pathway-specific regulatory genes. Specifically, three regulatory genes from BGC1.1 predicted to encode a non-ribosomal peptide/polyketide hybrid with unusual modifications were overexpressed (see Materials and methods).

The initial LC-MS analysis of extracts derived from the *Streptomyces* SL03 and SL06 mutant strains following both overexpression and knockout experiments, revealed no detectable changes in metabolite profiles compared to the wild type profiles. These results suggest that the targeted genetic modifications did not significantly impact the production of secondary metabolites.

**FIGURE 3:**
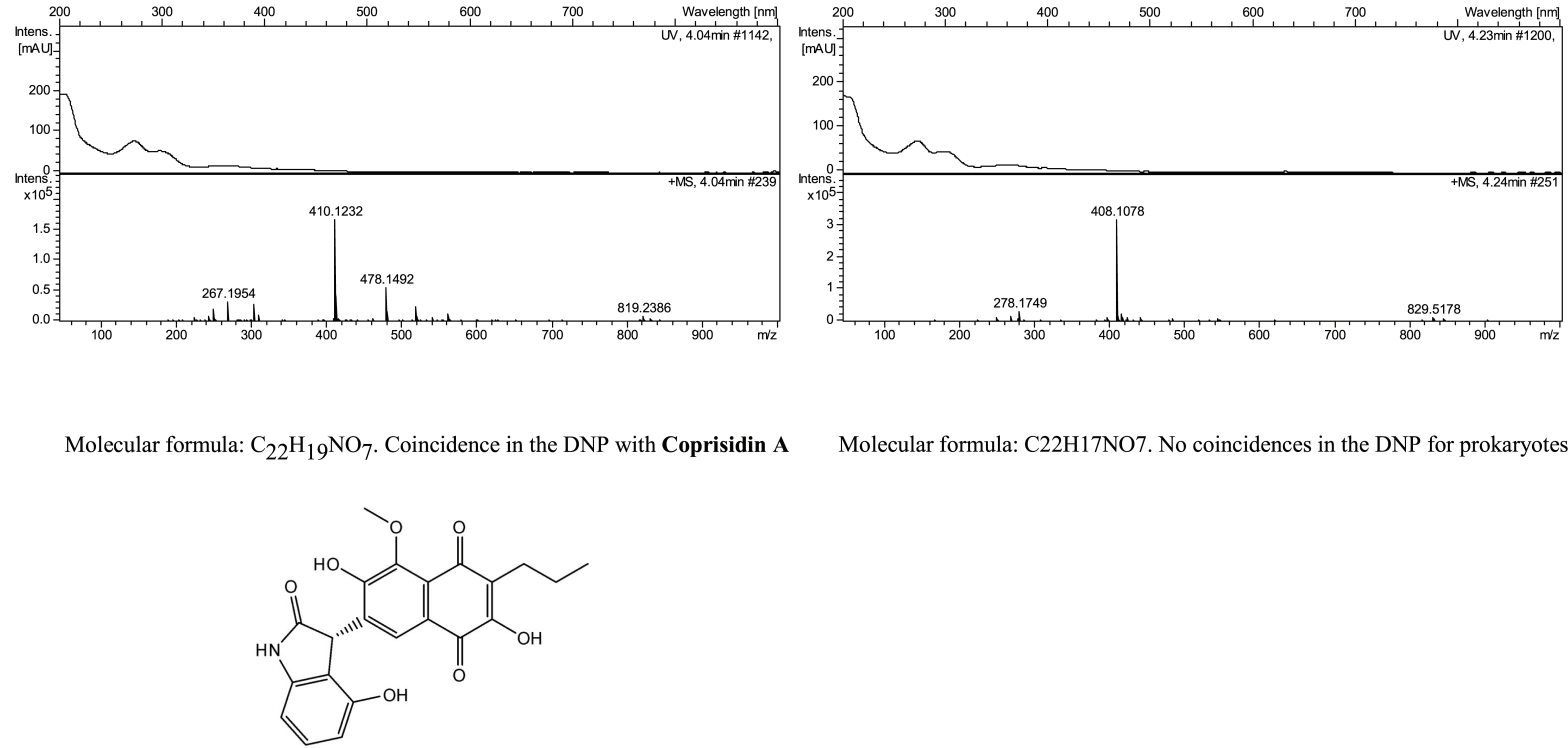
LC/MS results for *Streptomyces* sp. SL03 pinm02.

To further investigate the consequences of genetic manipulation, cultures were upscaled and extracted using the same methodology (see Materials and methods). LC/HRMS analysis of these extracts, conducted at Fundación MEDINA, combined with searches in the Chapman and Hall Dictionary of Natural Products (Dictionary of Natural Products 34.1 CRC Press, Taylor & Francis Group, an Informa Group company. (2025) revealed the presence of coprisidin A in ISP5 medium extracts together with a group of potentially novel natural products structurally related with the former according to their UV spectra, having molecular formulae of C_16_H_26_O_4_, C_16_H_26_O_5_, C_26_H_23_NO_8_, and C_22_H_17_NO_7_, the latter accounting for two hydrogen atoms less than coprisidin A (Figure 3 and Supplementary Figure S7). These compounds were exclusively detected in cultures overexpressing the SL03 transcriptional regulator 2 (see Supplementary Figure S6) grown in ISP5 medium, with no presence observed in the wild-type strain. Attempts to scale up the cultures, isolate and characterize the structure of these new derivatives of coprisidin A were unsuccessful due to low production levels.

An alternative cultivation and extraction method was employed and resulting extracts were analyzed using the Micro4All platform (see Materials and methods). This approach confirmed that overexpression did not induce the production of any new compounds beyond those already observed in the wild-type strain SL03.

Isolate SL06, which has shown antimicrobial activity in the previous experiments (Table 2) that cannot be linked to any identifiable compound, was subjected to gene inactivation within BGCs 2.2 and 2.19 (Supplementary Tables S5, S6; Supplementary Figure S5), none of which demonstrated similarity to previously identified BGCs. Although the antimicrobials activity was not abrogated in the resulting mutants, some differences in their metabolomes were observed compared to the wild type. Norharman was detected exclusively in the mutant strain SL06 pinm06, suggesting that the knockout of gene gtg2_5699 in BGC 2.19 (Supplementary Figure S5), may have caused unintended alterations in native gene expression, leading to the activation of norharman in a neighboring gene cluster. Moreover, mullinamide A and aminobacteriohopanetriol were present in both the wild-type and pinm06 strains but were absent in pinm04, where gtg2_455 in BGC 2.2 was knocked out. Conversely, tryptophol was detected in pinm04 but not in pinm06. Notably, the BGCs targeted for knockout did not correspond to the predicted biosynthetic clusters responsible for the production of these identified compounds, suggesting potential regulatory or compensatory interactions between biosynthetic pathways.

## Conclusion

This work presents the first detailed investigations of *Streptomyces* spp. isolated from freshwater sediments of the Laxenburg ponds (Austria). By integrating whole-genome sequencing with complementary metabolomic approaches, we show that these isolates harbor an unexpectedly large and diverse repertoire of biosynthetic gene clusters (BGCs). More than half of the BGCs identified across the six genomes lack close homologs in current reference databases, indicating the presence of pathways with the potential to yield structurally novel compounds, which may be developed into industrially and medically important products, e.g., pesticides, antimicrobials, immunosuppressants, anti-tumor agents etc. Metabolite profiling revealed that each isolate produces a distinct set of secondary metabolites, including siderophores, polyethers, polyketides, and peptide-derived compounds.

A comparison of ethanolic and methanolic extracts demonstrated the strong sensitivity of secondary metabolite detection to extraction methods and culture conditions, underscoring the value of employing multiple analytical strategies to capture different fractions of the metabolome. Two strains, SL03 and SL06, were selected for exploratory genetic manipulation aimed at activating poorly expressed clusters. Although overexpression of regulatory genes in SL03 did not lead to broad changes in metabolite production, it resulted in the emergence of coprisidin A and several related compounds that were absent in the wild-type strain. In SL06, targeted gene disruptions did not abolish production of the initially anticipated metabolites but instead altered the levels of unrelated compounds, pointing to a complex network of regulatory interactions among biosynthetic pathways.

Together, these results highlight both the promise and the complexity of mining silent or partially active BGCs in environmental *Streptomyces* spp. Overall, our findings underscore freshwater sediments as a valuable yet underexplored reservoir of *Streptomyces* with substantial biosynthetic potential. The strains described here offer multiple opportunities for future research, including heterologous expression of candidate BGCs and the development of cultivation strategies to induce the production of novel metabolites. Continued investigation of these isolates will advance our understanding of their ecological roles and facilitate the discovery of compounds with potential biotechnological or pharmaceutical applications.

## Materials and methods

### Sediment sample collection

On August 6th, 2020, sediment samples were manually collected from the Laxenburg ponds in Austria at the precise location (48° 3′ 57.33″ N, 16° 22′ 9.019″ E). A portion of these samples was promptly placed into sterile 50 mL Falcon tubes and cryogenically frozen in liquid nitrogen. Subsequently, the samples were preserved in a 20% sterile glycerol solution and stored at −80°C for future bacterial isolation.

### Bacterial isolation and growth conditions

Approximately 1–2 g of sediment were thawed and resuspended by vortexing in 3 mL of 9 g/L sterile NaCl solution. The resuspension was then subjected to serial dilutions (1:10^–1^ to 1:10^–4^) in the same sterile NaCl solution before being plated onto various nutrient agar media as described previously (Tocino-Márquez et al., 2025). To inhibit fungal growth, the media were supplemented with 50 μg/mL each of nystatin and cycloheximide, and, when necessary, 30 μg/mL nalidixic acid was added to suppress Gram-negative bacteria (Supplementary Table S1). In addition to media type, colony morphology, spore formation, and pigment production were used as criteria for isolating different *Streptomyces* species. Distinct colonies were carefully selected using a sterile toothpick and streaked onto their original growth media. To ensure pure cultures, colonies were repeatedly sub-cultured as needed. Pure cultures were harvested by scraping biomass from the plates and resuspending it in 2 mL cryotubes containing 20% sterile glycerol for long-term preservation at -80°C.

For genomic DNA isolation, 10–15 mL of selected liquid media (ISP2, liquid R2A or 2xYT) (Supplementary Table S2) were inoculated with 150 μL of the bacterial or spore suspensions. These cultures were incubated at 28°C with shaking at 200 rpm until sufficient biomass was produced for DNA extraction.

### DNA extraction and genome sequencing

Genomic DNA was extracted from six bacterial pellets using the PowerSoil Pro Kit (Qiagen, Germany) following the manufacturer’s instructions. DNA extraction and sequencing was performed at the Joint Microbiome Facility of the Medical University of Vienna and the University of Vienna under project ID JMF-2109–09.

Libraries were prepared for Oxford Nanopore Technologies (ONT) long read sequencing using the Rapid Barcoding kit SQK-RBK114.96 (Oxord Nanopore Technologies) following the manufacturers protocol. Barcoded libraries were loaded on a R10.4.1 flowcell (FLO-PRO114, Oxford Nanopore Technologies) and sequenced on a Promethion P24 (Oxford Nanopore Technologies). Basecalling was done using Guppy (v. 6.3.9) with the super accurate basecalling model. The basecalled reads were assembled using flye (v. 2.9.1, Kolmogorov et al., 2019) with “–nano-hq,” and polished once with medaka (v. 1.7.2). The model used was “r1041_e82_400bps_sup_g615.”

Assemblies were quality checked using QUAST (v. 5.2.0, Gurevich et al., 2013) and CheckM (lineage workflow, v. 1.2.3, Parks et al., 2015), and classified using GTDBtk (v. 2.4.0, Chaumeil et al., 2019).

### Construction of genome-based phylogenetic tree

Genome-based phylogenetic trees were computed using the Type strain Genome Server (TYGS) web platform (Leibniz Institute, DSMZ-German Collection of Microorganisms and Cell Cultures GmbH, Braunschweig, Germany). The closest type strain genomes were identified by comparing genomes of isolates from Laxenburg sediment samples (SL) with the TYGS database using the MASH algorithm (Ondov et al., 2016). For each genome, the ten type strains with the smallest MASH distances were selected. Additionally, a set of ten closely related type strains was identified by analyzing the 16S rRNA gene sequences extracted from SL genomes via RNAmmer (Lagesen et al., 2007), followed by BLAST analysis (Camacho et al., 2009) against the 16S rRNA gene sequences of the 19,367 type strains in the TYGS database. This method identified the top 50 matching type strains for each genome. Genome BLAST Distance Phylogeny (GBDP) (Meier-Kolthoff et al., 2013) was used to calculate precise genomic distances. The closest related strains and the genomes presented in this study were then uploaded to the TYGS platform to generate a final phylogenetic tree based on whole-genome data (see Figure 1). These results were cross-validated using genome data from closely related strains available in the NCBI database.

### Culture growth conditions for secondary metabolites production

For this study, three distinct fermentation media were used: Gause liquid medium, ISP5, and SM17 (Supplementary Table S2). Cultures were grown in 250 mL conical flasks, each containing 50 mL of the respective medium, and incubated at 28°C with continuous shaking at 200 rpm.

At the end of the fermentation process, the entire culture, including both cells and supernatant, was collected, rapidly frozen in a dry ice and ethanol mixture, and subsequently freeze-dried. Metabolites were extracted by adding methanol to the lyophilized biomass in a 1:1 ratio, followed by shaking at 150 rpm for 1 h at ambient temperature. After filtration, the resulting supernatants were transferred to 100 mL round-bottom flasks, and the solvent was evaporated under reduced pressure using a rotary evaporator. The remaining dried material was re-dissolved in 2.5 mL of methanol and stored at -20°C for future analysis. The high-resolution LC-MS analysis of these extracts was performed exactly as described previously (Vignolle et al., 2025).

A 15 mL culture of the microorganism was prepared in a 50 mL baffled flask and incubated under standard fermentation conditions. For extract preparation, 2 mL of the culture was transferred into a 15 mL centrifuge tube (Falcon) and mixed with 4 mL of ethanol. The tube was shaken for 1 h at 30°C, followed by centrifugation at 4,000 rpm for 8 min. After centrifugation, 125 μL of the supernatant was transferred to a well of a 96-well microtiter plate. This procedure was repeated for 80 individual cultures, filling 80 wells per plate, and multiple plates were prepared as needed. The plates were then left uncovered in a chemical fume hood for 24–48 h to dry, with drying time adjusted based on ambient humidity.

### Genetic manipulation of SL03 and SL06

To construct vectors for overexpression of regulatory genes gtg1_307, gtg1_339, and gtg1_351, located within Cluster 1.1 for NRPS-PKS (non-ribosomal peptide synthetase-polyketide synthase) from *Streptomyces* sp. SL03, respective genes were PCR-amplified using primers D0A3_307, D0A3_339 and DOA3_351.The primers included restriction sites *Not*I, *Eco*RI for gtg 1307, *Bam*HI *Eco*RI for gtg_1339 and *Not*I, *Eco*RI for gtg_1351 (Supplementary Table S8). The PCR-amplified fragments were digested and subsequently ligated with the vector pSET152-ermE*p, placing them under the control of constitutive strong promoter ermE*p. The validated constructs were then transferred to the wild-type strain *Streptomyces* sp. SL03 via conjugation.

For gene knock-out experiments, two type I PKS genes, specifically, gtg2_455 and gtg2_5699, located within BGC2.2 and 2.19 in *Streptomyces* sp. SL06, were targeted. Fragments of target genes were PCR-amplified using primers D0C7_455 and D0C7_5699 (Supplementary Table S8) containing *Eco*RI and *Hin*dIII restriction sites. After digestion, each *Eco*RI-*Hin*dIII fragment was ligated with 3kb fragment from pSOK201 suicidal vector. Knock-out vectors were introduced into *Streptomyces* sp. SL06 wild type strain via conjugation.

### Biosynthetic gene cluster identification and characterization

To identify BGCs, genomic analyses were carried out using the antiSMASH 7.0 platform (Blin et al., 2023). GenBank-formatted genome files were uploaded to the antiSMASH server, with detection parameters set to a relaxed threshold to enhance cluster identification. Several analytical tools, such as KnownClusterBlast, ClusterBlast, MiBiG cluster comparison, and ActiveSiteFinder, were used to improve the accuracy of BGC predictions. The antiSMASH tool executes a gene identification algorithm, locating BGCs based on the presence of core enzymes responsible for secondary metabolite biosynthesis. Inclusion of KnownClusterBlast, ClusterBlast, and MiBiG functions facilitates comparative analysis against publicly available genomic data, aiding in discovering potentially novel bioactive compounds. Our analysis prioritized BGCs encoding NRPS, PKS, RiPPs, as well as complex or hybrid gene clusters.

LC/HRMS analyses performed at Fundación MEDINA used an Agilent 1200RR HPLC coupled to a Bruker maXis q-TOF analyzer operated in positive ESI mode. Chromatographic and ionization conditions were described previously.

### Bioactivity testing: disk diffusion/drop assays

Susceptibility testing was performed using the agar disc diffusion technique to assess the antimicrobial properties of the bacterial extracts. The activity of the extracts was tested against a range of microbial strains (Supplementary Table S3), including *Escherichia coli* DH5α, *Pseudomonas putida* KT2440, *Bacillus subtilis* DSMZ 10, *Micrococcus luteus* DSMZ 1790, *Staphylococcus carnosus* DSMZ 20501, and *Saccharomyces cerevisiae* BY4742. For each assay, agar plates were prepared using Lennox agar (LA), tryptic soy agar (TSA), and yeast extract peptone dextrose (YPD) media. A 150 μL aliquot of the respective test organism, derived from either a 20% glycerol microbial suspension or a freshly prepared seeding culture grown in LB or TSB broth, was evenly spread onto the agar surface. After the inoculated plates were dried, sterile 9 mm filter paper discs (Whatman, GE Healthcare Life Sciences, United States) impregnated with 50 μL of the crude methanolic extract were placed onto the surface. The discs were previously dried under sterile conditions to ensure the complete evaporation of the solvent. The plates were then incubated at the appropriate temperature (28°C or 37°C, depending on the organism, as detailed in Supplementary Table S3). Following the incubation period, the zones of growth inhibition around each disc were measured to quantify the extract’s antimicrobial efficacy.

## Data Availability

The datasets presented in this study can be found in online repositories. The names of the repository/repositories and accession number(s) can be found at: https://www.ncbi.nlm.nih.gov/, PRJNA1380313.

## Footnotes

1
https://mibig.secondarymetabolites.org
