# Supplementary material for: Unveiling the genomes and secondary metabolomes of Streptomyces spp. from freshwater sediments
Source: Front Microbiol. 2026 Apr 20;17:1793713. doi: 10.3389/fmicb.2026.1793713 (PMC13136089; doi:10.3389/fmicb.2026.1793713)
Supplement: Supplementary file 1 [file Data_Sheet_1.doc]

**Supplementary Materials**

**Unveiling the Genomes and Secondary Metabolomes of *Streptomyces* spp. from Freshwater Sediments**

Inmaculada Tocino-Márquez^1,5^, Martin Zehl^2,7^, Jovana Batajic^1^, Joana Séneca^3,4^, Petra Pjevac^3,4^, José Murillo-Alba^6^, Jesús Martín^6^, Olga N. Sekurova^1^, Sergey B. Zotchev^1^*

^1^Department of Pharmaceutical Sciences, Division of Pharmacognosy, University of Vienna, 1090 Vienna, Austria

^2^Department of Analytical Chemistry, Faculty of Chemistry, University of Vienna, 1090 Vienna, Austria

^3^Centre for Microbiology and Environmental Systems Science, University of Vienna, 1030 Vienna, Austria

^4^Joint Microbiome Facility of the Medical University of Vienna and the University of Vienna, 1030 Vienna, Austria

^5^Doctoral School in Microbiology and Environmental Science, University of Vienna, 1030 Vienna, Austria

^6^Fundación MEDINA, 18016, Armilla, Granada, Spain

^7^Institute of Science and Technology Austria (ISTA), 3400 Klosterneuburg, Austria

**Table S1.** Nutrient media recipes [g/L] for bacterial isolation.

| **Ingredients [g/L]** | **SCA** | **AIA** | **ISP4**  **agar** |
| --- | --- | --- | --- |
| Starch | 10 | - | 10.0 |
| Glycerol | - | - | - |
| Casein peptone | 0.30 | - | - |
| Sodium caseinate |  | 2.0 | - |
| Sodium propionate |  | 4.0 | - |
| L-Asparagine | - | 0.1 | - |
| K_2_HPO_4_ x 3H_2_O | 2.0 | 0.5 | 1.0 |
| KNO_3_ | 2.0 |  |  |
| CaCO_3_ | 0.02 | - | 2.0 |
| NaCl | 2.0 | - | - |
| KCl | - | - | 1.0 |
| MnCl_2_ | - | - | 0.001 |
| (NH_4_)_2_SO_4_ | - | - | 2.0 |
| MgSO_4_ x 7H_2_O | 0.05 | 0.1 | 1.0 |
| FeSO_4_ x 7H_2_O | 0.01 | 0.001 | 0.001 |
| ZnSO_4_ x 7H_2_O | - | - | 0.001 |
| Nystatin | + | + | + |
| Cycloheximide | + | + | + |
| Agar | 18.0 | 15.0 | 20.0 |
| pH | 7.0 | 8.1 | 7.2 |

**Table S2.** Nutrient media recipes [g/L] for bacterial growth and fermentation

| **Ingredients [g/L]** | **SFM** | **2xYT** | **SM17** | **R2A** | **ISP2** | **ISP5** | **Gause** |
| --- | --- | --- | --- | --- | --- | --- | --- |
| Glucose | - | - | 2.0 | 0.5 | 4.0 |  | - |
| Starch | - | - | - | 0.5 |  |  | 20.0 |
| Glycerol | - | - | 20.0 | - |  | 10.0 | - |
| Malt extract | - | - | - | - | 10.0 |  | - |
| Yeast extract |  |  |  |  | 4.0 |  |  |
| Peptone | - | - | 5.0 | - |  |  | - |
| Soluble starch | - | - | 2.0 | - |  |  | - |
| Soy flour | 20.0 | - | 5.0 | - |  |  | - |
| Soy peptone | 5.0 | - | - | - |  |  | - |
| Proteose peptone | - | - | - | 0.5 |  |  | - |
| Tryptone | - | 16.0 | - | - |  |  | - |
| Casamino acids | - | - | - | 0.5 |  |  | - |
| Yeast extract | - | 10.0 | 5.0 | 0.5 |  |  | - |
| Na-pyruvate | - | - | - | 0.3 |  |  | - |
| CaCO_3_ | - | - | 2.0 | - |  |  | - |
| NaCl | - | 5.0 | 5.0 | - |  |  | 0.5 |
| K_2_HPO_4_ | - | - | - | 0.3 |  | 1000 ml | 0.5 |
| MgSO_4_ | - | - | - | 0.05 |  |  | 0.5 |
| FeSO_4_ | - | - | - | - |  | 1.0 | 18.0 |
| MnSO_4_ | - | - | - | - |  | 1.0 | - |
| KNO_3_ |  |  |  |  |  |  | 1.0 |
| ZnSO_4_ |  |  |  |  |  | 1.0 |  |
| Agar | 20.0 | - | - | 15 | 20 | 20 | 15.0 |
| Tap water | -/+ | + | + | -/+ | + | + | -/+ |
| pH | 7.0 | 7.2 | 6.4 | 7.2 | 7.2 | 7.0 | 7.4 |

**Table S3.** Test organisms and growth conditions for bioactivity testing.

| **Test organism** | **Medium** | **Growth conditions** |
| --- | --- | --- |
| *Bacillus subtilis* DSMZ 10 | TSB | 28°C, overnight |
| *Escherichia coli* DH5α | LB | 37°C, overnight |
| *Micrococcus luteus* DSMZ 1790 | TSB | 28°C, overnight |
| *Pseudomonas putida* KT2440 | LB | 37°C, overnight |
| *Saccharomyces cerevisiae* BY4743 | YPD | 28°C, overnight |
| *Staphylococcus carnosus* DSMZ 20501 | TSB | 37°C, overnight |

**Figure S1.** Complete inhibition observed in ISP5 medium extract from strain SL02 and partial inhibition from SM17 and Gause’s extract against *M. luteus*.


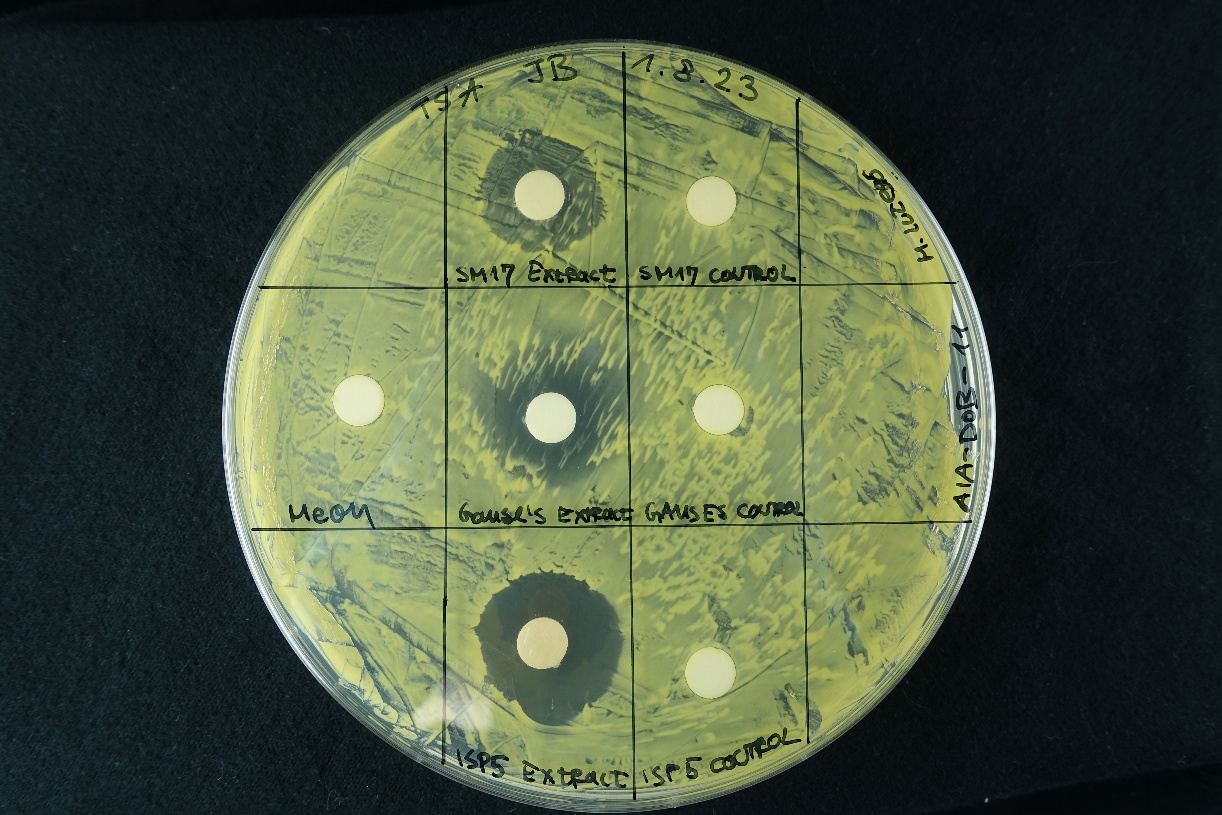


**Figure S2.** Partial inhibition of the strain SL06 in Gause medium against *E. coli*


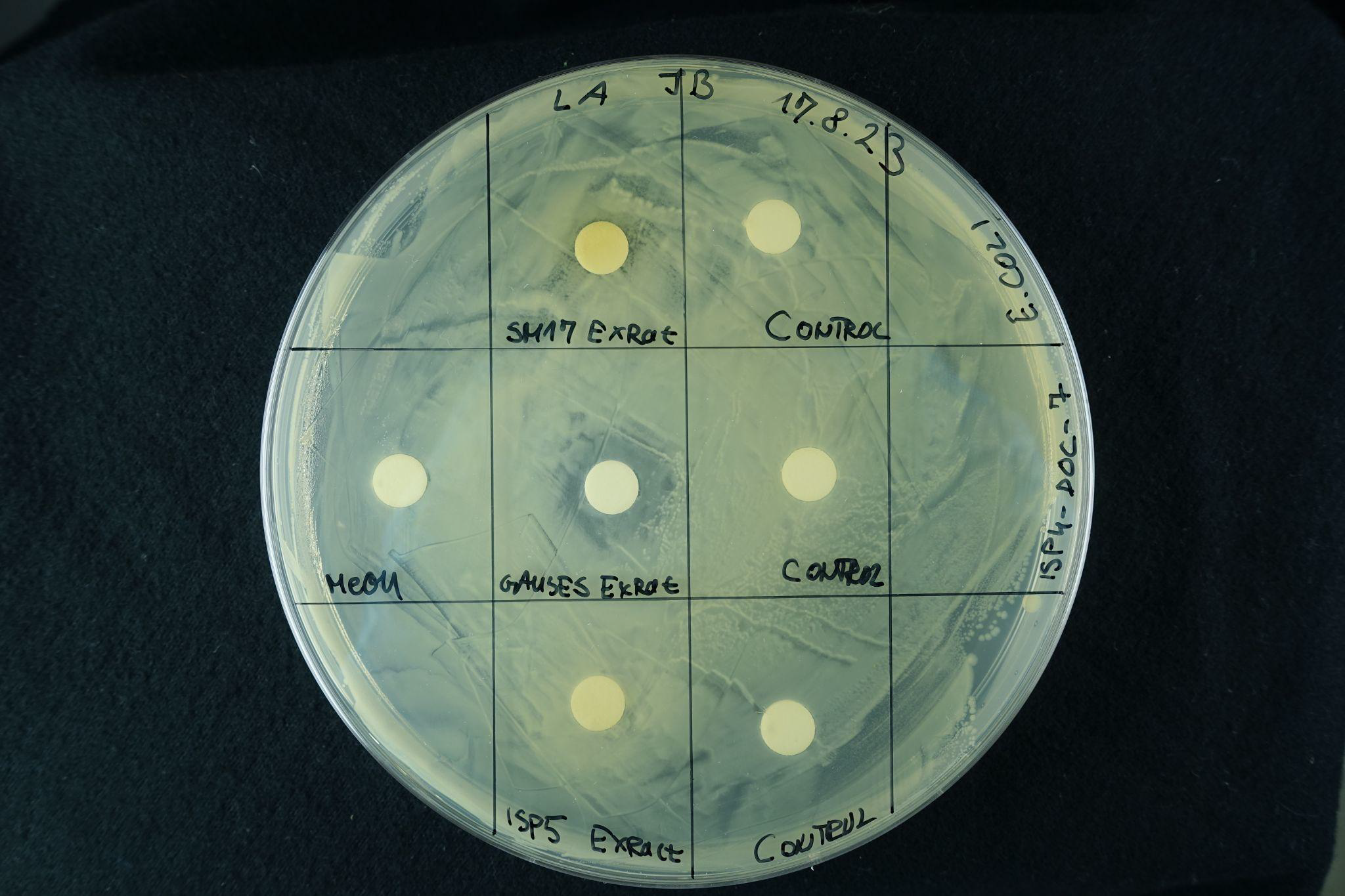


**Figure S3.** Partial inhibition of the strain SL05 in Gause medium against *E. coli*


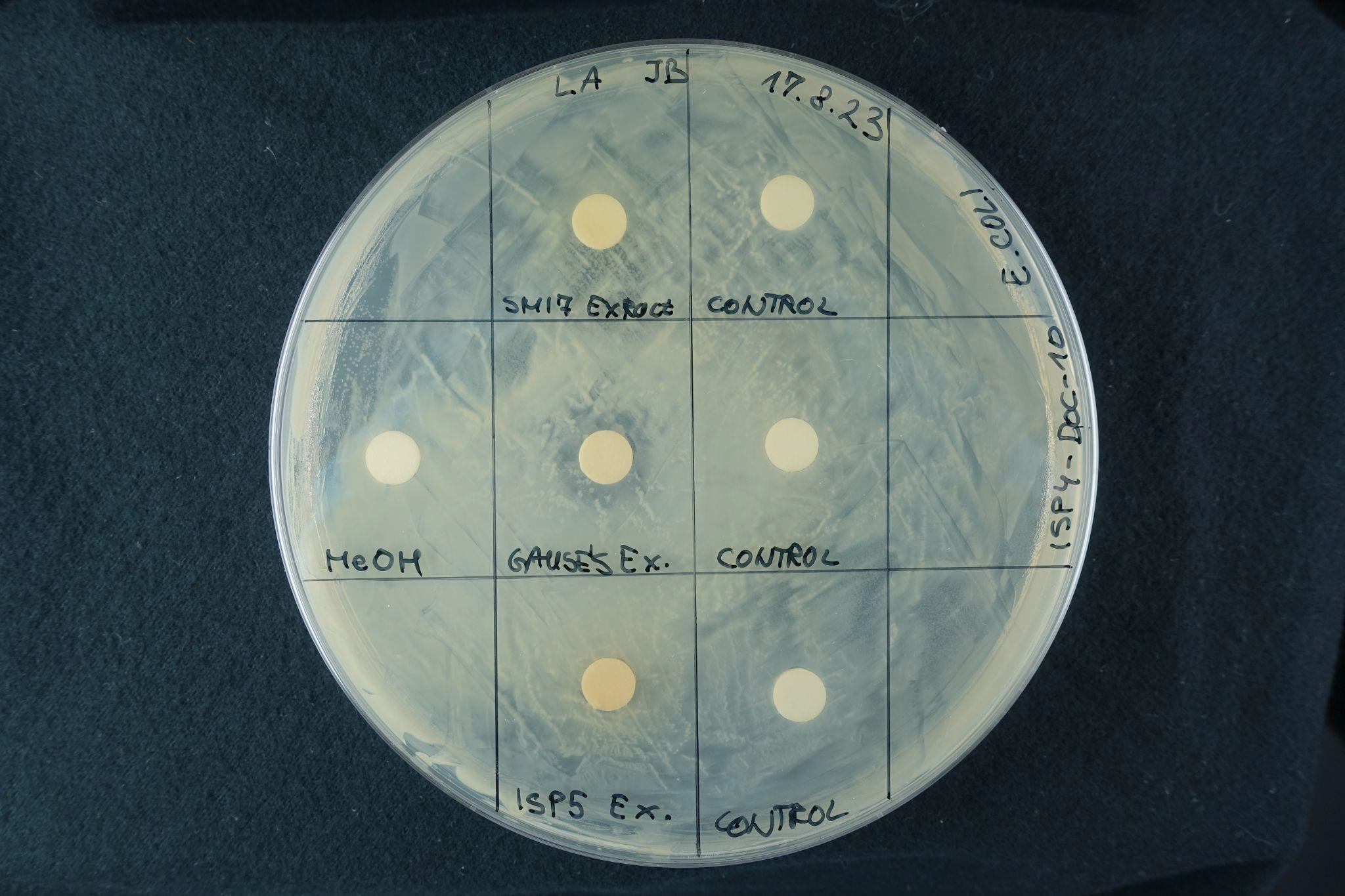


**Figure S4.** Complete inhibition observed in the test of the 3 extracts (SM17, Gause, and ISP5) from strain SL04 against *S. carnosus.*


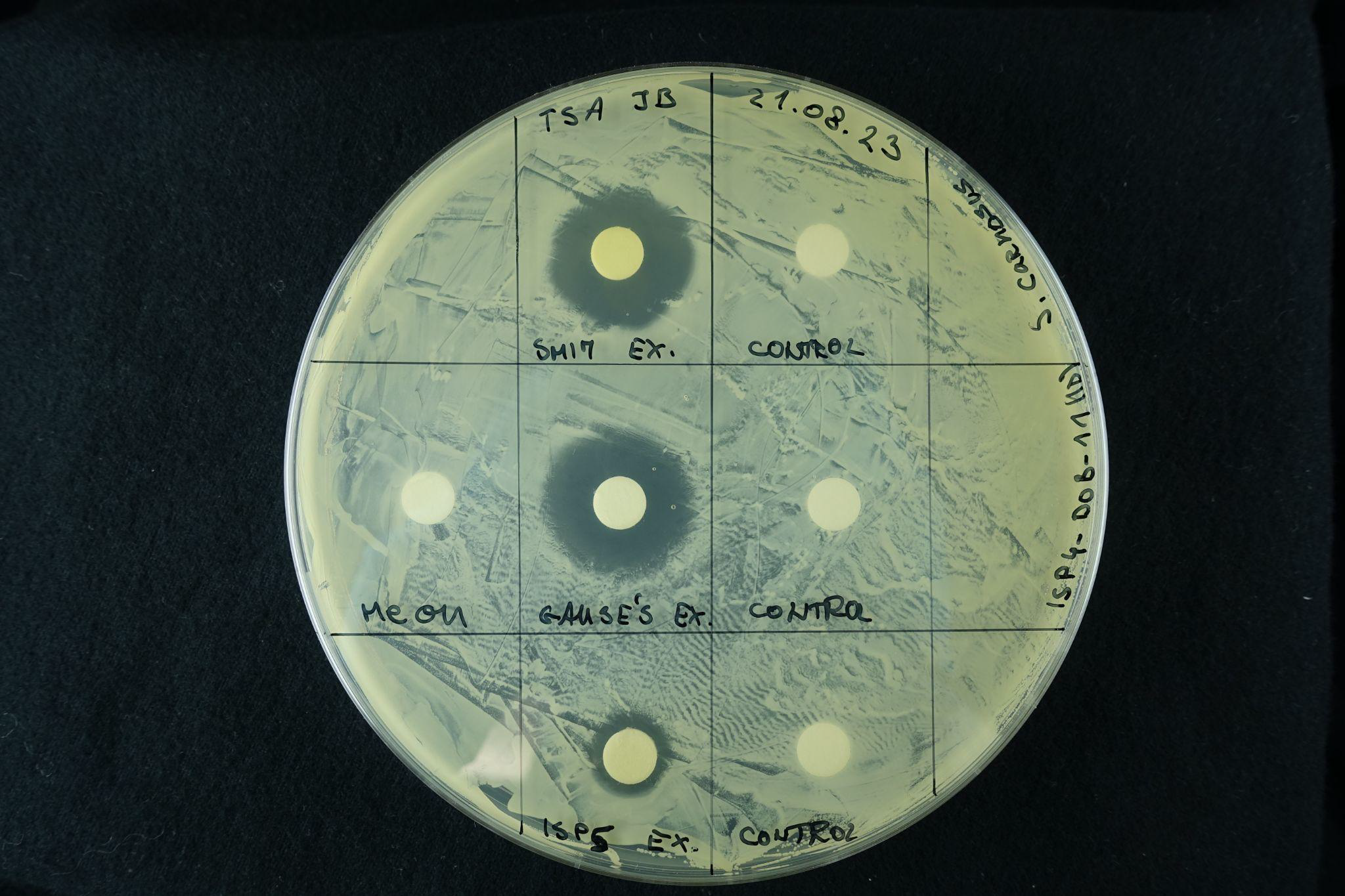


| **Strain** | **No. of biosynthetic gene clusters** | | | | | | | | | | | | |
| --- | --- | --- | --- | --- | --- | --- | --- | --- | --- | --- | --- | --- | --- |
|  | **Betalactone** | **HSL** | **Hydrogen-cyanide** | **NRPS** | **NRPS-PKS** | **PKS** | **PKS I** | **PKS II** | **PKS III** | **RIPP** | **Siderophore** | **Terpene** | **Others** |
| **SL01** |  |  | 1 | 7 | 5 |  | 2 | 1 | 1 | 4 | 6 | 7 | 4 |
| **GCA_014650955** |  |  | 1 | 6 | 5 |  | 9 | 1 | 1 | 7 | 6 | 6 | 4 |
| **SL02** |  |  |  | 3 | 5 | 1 | 1 | 1 |  | 5 | 1 | 6 | 4 |
| **GCA_001700505** |  |  |  | 8 | 8 | 1 | 19 | 1 |  | 6 | 1 | 6 | 4 |
| **SL03** |  |  |  | 3 | 2 |  | 1 | 2 | 1 | 5 | 2 | 6 | 5 |
| **GCF_000717245** |  |  |  | 4 | 2 |  | 2 | 2 | 1 | 5 | 2 | 6 | 4 |
| **SL04** | 1 | 1 |  | 9 | 3 | 2 | 6 | 2 |  | 5 | 2 | 8 | 7 |
| **GCA_005405925** | 2 |  |  | 9 | 3 | 2 | 7 | 2 | 1 | 4 | 2 | 8 | 5 |
| **SL05** |  |  |  | 4 | 2 |  |  | 1 |  | 6 | 2 | 4 | 1 |
| **GCF_004195745** |  |  |  | 6 | 3 |  | 2 |  | 1 | 7 | 2 | 4 | 1 |
| **SL06** |  |  |  | 3 | 2 | 1 | 2 | 2 | 1 | 2 | 2 | 7 | 4 |
| **GCA_039543405** |  |  | 1 | 9 |  |  | 2 | 1 | 3 | 8 | 2 | 6 | 6 |

**Table S4.** Abundance of BGCs of SL bacterial detected by antiSMASH 7.1.0 compared to their closest known strains from online databases.

**Table S5.** List of ethanolic extracts analyzed for secondary metabolite profiling using Micro4All Molecules Gateway (Naicons).

| **Position** | **Extract ID** | **Medium** | **Type Strain** | **Taxonomy** | **Solvent** | **Culture volume** | **µl final extract** |
| --- | --- | --- | --- | --- | --- | --- | --- |
| **1** | **CONTROL SM17** | SM17 | - | - | Ethanol | 30 ml | 120 |
| **2** | **CONTROL ISP5** | ISP5 | - | - | Ethanol | 30 ml | 120 |
| **3** | **CONTROL GAUSE** | GAUSE | - | - | Ethanol | 30 ml | 120 |
| **4** | **SL04** | SM17 | Wild type | *Streptomyces* sp. | Ethanol | 30 ml | 120 |
| **5** | **SL04** | ISP5 | Wild type | *Streptomyces* sp. | Ethanol | 30 ml | 120 |
| **6** | **SL04** | GAUSE | Wild type | *Streptomyces* sp. | Ethanol | 30 ml | 120 |
| **7** | **SL03** | SM17 | Wild type | *Streptomyces* sp. | Ethanol | 30 ml | 120 |
| **8** | **SL03** | ISP5 | Wild type | *Streptomyces* sp. | Ethanol | 30 ml | 120 |
| **9** | **SL03** | GAUSE | Wild type | *Streptomyces* sp. | Ethanol | 30 ml | 120 |
| **10** | **SL06** | SM17 | Wild type | *Streptomyces* sp. | Ethanol | 30 ml | 120 |
| **11** | **SL06** | ISP5 | Wild type | *Streptomyces* sp. | Ethanol | 30 ml | 120 |
| **12** | **SL06** | GAUSE | Wild type | *Streptomyces* sp. | Ethanol | 30 ml | 120 |
| **13** | **SL05** | SM17 | Wild type | *Streptomyces* sp. | Ethanol | 30 ml | 120 |
| **14** | **SL05** | ISP5 | Wild type | *Streptomyces* sp. | Ethanol | 30 ml | 120 |
| **15** | **SL05** | GAUSE | Wild type | *Streptomyces* sp. | Ethanol | 30 ml | 120 |
| **16** | **SL01** | SM17 | Wild type | *Streptomyces* sp. | Ethanol | 30 ml | 120 |
| **17** | **SL01** | ISP5 | Wild type | *Streptomyces* sp. | Ethanol | 30 ml | 120 |
| **18** | **SL01** | GAUSE | Wild type | *Streptomyces* sp. | Ethanol | 30 ml | 120 |
| **19** | **SL02** | SM17 | Wild type | *Streptomyces* sp. | Ethanol | 30 ml | 120 |
| **20** | **SL02** | ISP5 | Wild type | *Streptomyces* sp. | Ethanol | 30 ml | 120 |
| **21** | **SL02** | GAUSE | Wild type | *Streptomyces* sp. | Ethanol | 30 ml | 120 |

| **Position** | **Extract ID** | **Medium** | **Type Strain** | **Taxonomy** | **Solvent** | **Culture volume** | **µl final extract** |
| --- | --- | --- | --- | --- | --- | --- | --- |
| **22** | **SL03 pINM01** | SM17 | Mutant pINM01 | *Streptomyces* sp. | Ethanol | 30 ml | 120 |
| **23** | **SL03 pINM01** | ISP5 | Mutant pINM01 | *Streptomyces* sp. | Ethanol | 30 ml | 120 |
| **24** | **SL03 pINM01** | GAUSE | Mutant pINM01 | *Streptomyces* sp. | Ethanol | 30 ml | 120 |
| **25** | **SL03 pINM02** | SM17 | Mutant pINM02 | *Streptomyces* sp. | Ethanol | 30 ml | 120 |
| **26** | **SL03 pINM02** | ISP5 | Mutant pINM02 | *Streptomyces* sp. | Ethanol | 30 ml | 120 |
| **27** | **SL03 pINM02** | GAUSE | Mutant pINM02 | *Streptomyces* sp. | Ethanol | 30 ml | 120 |
| **28** | **SL03 pINM03** | SM17 | Mutant pINM03 | *Streptomyces* sp. | Ethanol | 30 ml | 120 |
| **29** | **SL03 pINM03** | ISP5 | Mutant pINM03 | *Streptomyces* sp. | Ethanol | 30 ml | 120 |
| **30** | **SL03 pINM03** | GAUSE | Mutant pINM03 | *Streptomyces* sp. | Ethanol | 30 ml | 120 |
| **31** | **SL06 pINM04** | SM17 | Mutant pINM04 | *Streptomyces* sp. | Ethanol | 30 ml | 120 |
| **32** | **SL06 pINM04** | ISP5 | Mutant pINM04 | *Streptomyces* sp. | Ethanol | 30 ml | 120 |
| **33** | **SL06 pINM04** | GAUSE | Mutant pINM04 | *Streptomyces* sp. | Ethanol | 30 ml | 120 |
| **34** | **SL06 pINM06** | SM17 | Mutant pINM06 | *Streptomyces* sp. | Ethanol | 30 ml | 120 |
| **35** | **SL06 pINM06** | ISP5 | Mutant pINM06 | *Streptomyces* sp. | Ethanol | 30 ml | 120 |
| **36** | **SL06 pINM06** | GAUSE | Mutant pINM06 | *Streptomyces* sp. | Ethanol | 30 ml | 120 |

**Table S6.** Secondary metabolites identified using the Micro4All Molecules Gateway (Naicons).

| **Confidence** | **Extracts position** | **NAME** | **STRAIN, BGC** | **REFERENCE** |
| --- | --- | --- | --- | --- |
| 7 | ['10', '34', '31'] | Megalochelin | SL06, BGC2.24 | Vind K et al., 2023 |
| 7 | ['11'] | Megalochelin linear form | SL06, BGC2.24 | Vind K et al., 2023 |
| 7 | ['13', '16'] | Deferoxamine | SL05, BGC1.14; SL01, BGC2.16 | García-Martín J et al., 2024; Arshadi Z et al., 2021; Gerber NN, 1971 |
| 7 | ['16'] | Undecylprodigiosin | SL01, BGC2.5 | Chaeyoung Lee et al., 2024; Ramesh C et al., 2021; Feitelson J S et al., 1985 |
| 7 | ['18', '16'] | Streptorubin B | SL01, BGC2.5 | Marshall AP et al. 2020; Withall DM et al., 2015; Haynes SW et al., 2011 |
| 7 | ['4', '5', '6'] | Niphimycin | SL04, BGC3.5 | Huang Y et al. 2023; Chen Y et al. 2022; Againa S et al. 1975 |
| 7 | ['18', '4', '5', '21', '7'] | Scopafungin | SL04, BGC3.6 | Nair S, 2020; Li Z 2019; Trejo-Estrada SR, Paszczynski A and Crawford DL, 1998 |
| 7 | ['4', '5', '6'] | niphimycin D | SL04, BGC3.5 | Yuanyuan Hu et al. 2018 |
| 7 | ['4', '13', '16'] | nocardamin | SL04, BGC3.22; SL05, BGC1.14; SL01, BGC2.16 | Mahmud F et al. 2022; Lopez JAV et al. 2019; Kalinovskaya et al. 2011; Yang CC and Leong J. 1989 |
| 7 | ['4'] | Nigericin | SL04, BGC3.6 | Zhu et al., 2022; Gao G et al., 2021 |
| 7 | ['13', '15'] | Antimycin A15 | SL05, BGC1.20 | Braddock AA and Theodorakis EA, 2019 |
| 7 | ['13'] | Antimycin A1a | SL05, BGC1.20 | Braddock AA and Theodorakis EA, 2019 |
| 7 | ['13', '15', '16'] | Antimycin A17 | SL05, BGC1.20; SL01, BGC2.1 | Braddock AA and Theodorakis EA, 2019 |
| 7 | ['13'] | Antimycin A20 | SL05, BGC1.20 | Braddock AA and Theodorakis EA, 2019 |
| 7 | ['13', '15', '16'] | Antimycin A19 | SL05, BGC1.20; SL01, BGC2.1 | Braddock AA and Theodorakis EA, 2019 |
| 7 | ['13'] | Antimycin A7b | SL05, BGC1.20 | Braddock AA and Theodorakis EA, 2019 |
| 7 | ['13'] | Antimycin_A2 | SL05, BGC1.20 | Braddock AA and Theodorakis EA, 2019 |
| 7 | ['13'] | Antimycin_A4A | SL05, BGC1.20 | Braddock AA and Theodorakis EA, 2019 |
| 7 | ['13'] | Alteramide B | SL05, BGC1.2 | Ding Y et al., 2016; Tang B et al., 2021 |
| 7 | ['9', '27', '28', '13', '30', '7', '15', '24'] | Alteramide A | SL03, BGC1.1; SL05, BGC1.2 | Shigemori, H et al., 1992 |
| 7 | ['9', '27', '28', '13', '30', '7', '15', '24'] | Maltophilin | SL03, BGC1.1; SL05, BGC1.2 | Jakobi M et al., 1996 |
| 7 | ['28', '13', '7', '15'] | Ikarugamycin_epoxide | SL03, BGC1.1; SL05, BGC1.2 | Bertasso M et al., 2003 |
| 7 | ['18', '5', '21', '6', '15'] | Grisorixin | SL04, BGC3.6 | Leulmi N et al., 2019; Gachon et al., 1975 |
| 7 | ['9', '33', '26', '34', '11', '19', '35', '4', '36', '5', '13', '21', '6', '24', '32'] | Nigericin like | SL04, BGC3.6; SL06, BGC2.2 | Zhu et al., 2022; Gao G et al., 2021 |
| 7 | ['13', '14', '15'] | Surugamide_A | SL05, BGC1.12 | Takada K et al., 2013; Matsuda K et al., 2019 |
| 7 | ['10', '34', '19', '31'] | ENTEROBACTIN-like | SL06, BGC2.21; SL02, BGC2.4 | Raymond KN et al., 2003 |
| 7 | ['13', '15'] | Levorin A2 | SL05, BGC1.19 | Szczeblewski et al., 2017, Shenin IuD, 1987 |
| 7 | ['13', '15'] | Levorin A3 | SL05, BGC1.19 | Borzyszkowska-Bukowska J et al., 2019 |
| 7 | ['13'] | Levorin_analog_912 | SL05, BGC1.19 | Iarobkova ND, 1970 |
| 7 | ['13'] | Acyl-Desferrioxamine_C12 | SL05, BGC1.14 | Parker JB et al., 2023; Ramadhan MIA et al., 2023; Fujisawa K. et al., 2022; Farmakis et al., 2021 |
| 6 | ['27', '12', '36', '21', '29', '30', '24'] | "Aminobacteriohopanetriol;_Bacteriohopaneaminotriol; | SL03, BGC1.6 | Zhang H et al., 2022; Liu W et al., 2014 |
| 6 | ['13'] | ANTIMYCIN_A6 | SL05, BGC1.20 | Ogita M et al., 2009 |
| 6 | ['13', '16'] | Desferri-ferrioxamine_H | SL05, BGC1.14; SL01, BGC2.16 | Parker JB et al., 2023; Ramadhan MIA et al., 2023; Fujisawa K. et al., 2022; Farmakis et al., 2021 |
| 6 | ['13'] | Proferrioxamine-A1 | SL05, BGC1.14 |  |
| 6 | ['4', '28', '22', '7'] | COELICHELIN | SL04, BGC3.3; SL03, BGC1.2 | Barona-Gómez F et al., 2006; Challis Gl and Ravel J, 2000 |
| 5 | ['25', '19', '28', '13', '7'] | Schizokinen | SL02, BGC2.11; SL03, BGC1.9 | Chuljerm H et al., 2019; Mullis KB et al., 1971; |
| 6 | ['10'] | BONACTIN | SL06, most likely BGC2.2 | Thiyagarajamoorthy DK et. al., 2018; Schumacher RW et al., 2003 |

**Table S7.** LC-MS based analyses and putative identification of secondary metabolites from methanolic extracts.

| **#** | **Strain** | **Rt** | ***m/z*** |  |  |  | **Sum formula** | ***m/z*** | **Δ*m/z*** | **Tentative ID** | **BGC** |
| --- | --- | --- | --- | --- | --- | --- | --- | --- | --- | --- | --- |
|  |  | **[min]** | **[M+H]+** | **[M+2H]2+** | **[M+3H]3+** | **[M+Na]+** | **(proposed)** | **calcd.** | **[ppm]** | |  |
|  | SL04 | 12,4 | 601,3559 |  |  |  | C27H48N6O9 | 601,3556 | -0,6 | Desferrioxamine E |  |
|  | SL04 | 23,2 | 1056,7318 |  |  |  | C56H101N3O15 | 1056,7305 | -1,2 | Amycin B |  |
|  | SL04 | 23,3 | 1142,7320 |  |  |  | C59H103N3O18 | 1142,7309 | -0,9 | Niphimycin C or isomer |  |
|  | SL04 | 23,5 | 1228,7315 |  |  |  | C62H105N3O21 | 1228,7313 | -0,1 | Niphimycin D or E |  |
|  | SL04 | 24,3 | 1228,7316 |  |  |  | C62H105N3O21 | 1228,7313 | -0,2 | Niphimycin D or E |  |
|  | SL03 | 2,9 | 229,1294 |  |  |  | C9H16N4O3 | 229,1295 | 0,6 | Deoxyguanidinoproclavaminic acid? | 1.5 |
|  | SL03 | 6,2 | 421,1934 |  |  |  | C16H28N4O9 | 421,1929 | -1,2 | Schizokinen | 1.9 |
|  | SL03 | 23,5 | 511,2808 |  |  |  | C29H38N2O6 | 511,2803 | -1,1 | Alteramide A or isomer | 1.1 |
|  | SL03 | 21,0 | 655,2766 |  |  |  | C36H38N4O8 | 655,2762 | -0,5 | Coproporphyrin |  |
|  | SL06 | 4,2 |  |  |  | 625,1585 | C22H34O19 | 625,1586 | 0,2 | Ribocitrin |  |
|  | SL06 | 6,4 | 242,0662 |  |  |  | C10H11NO6 | 242,0659 | -1,3 | *N*-(2,3-dihydroxybenzoyl)serine | 2.21 |
|  | SL06 | 9,7 | 1447,6451 | 724,3264 |  |  | C60H90N18O24 | 1447,6448 | -0,2 | New siderophore | 2,2 |
|  | SL06 | 11,6 | 1449,6610 | 725,3341 |  |  | C60H92N18O24 | 1449,6605 | -0,4 | New siderophore | 2,2 |
|  | SL06 | 12,0 | 292,0928 |  |  |  | C13H13N3O5 | 292,0928 | 0,1 | PDE-I |  |
|  | SL06 | 12,9 | 169,0859 |  |  |  | C9H12O3 | 169,0859 | 0,4 | Germicidin D or isomer | 2.20 |
|  | SL06 | 13,9 | 228,1385 |  |  |  | C15H17NO | 228,1383 | -0,8 | Dimethylallylindole congener | 2,2 |
|  | SL06 | 15,0 | 183,1017 |  |  |  | C10H14O3 | 183,1016 | -0,7 | Germicidin B | 2.20 |
|  | SL06 | 15,1 | 183,1016 |  |  |  | C10H14O3 | 183,1016 | -0,3 | Germicidin C | 2.20 |
|  | SL06 | 15,3 | 183,1016 |  |  |  | C10H14O3 | 183,1016 | -0,1 | Isogermicidin B | 2.20 |
|  | SL06 | 16,9 | 197,1172 |  |  |  | C11H16O3 | 197,1172 | 0,1 | Germicidin A | 2.20 |
|  | SL06 | 17,2 | 197,1172 |  |  |  | C11H16O3 | 197,1172 | 0,0 | Isogermicidin A | 2.20 |
|  | SL06 | 18,8 | 211,1329 |  |  |  | C12H18O3 | 211,1329 | 0,0 | New germicidin | 2.20 |
|  | SL06 | 19,7 | 243,1491 |  |  |  | C15H18N2O | 243,1492 | 0,5 | Dimethylallylindole-3-acetaldoxime | 2,2 |
|  | SL06 | 19,7 | 246,1490 |  |  |  | C15H19NO2 | 246,1489 | -0,6 | Dimethylallylindole congener | 2,2 |
|  | SL06 | 22,4 | 230,1540 |  |  |  | C15H19NO | 230,1539 | -0,3 | Prenyltryptophol | 2,2 |
|  | SL06 | 31,8 |  | 1070,0874 |  |  |  |  |  | PepX |  |

| **#** | **Strain** | **Rt** | ***m/z*** |  |  |  | **Sum formula** | ***m/z*** | **Δ*m/z*** | **Tentative ID** | **BGC** |
| --- | --- | --- | --- | --- | --- | --- | --- | --- | --- | --- | --- |
|  |  | **[min]** | **[M+H]+** | **[M+2H]2+** | **[M+3H]3+** | **[M+Na]+** | **(proposed)** | **calcd.** | **[ppm]** | |  |
|  | SL05 | 9,5 | 561,3611 |  |  |  | C25H48N6O8 | 561,3606 | -0,8 | Desferrioxamine B | 1.14 |
|  | SL05 | 11,0 | 573,3247 |  |  |  | C25H44N6O9 | 573,3243 | -0,7 | Desferrioxamine X7 | 1.14 |
|  | SL05 | 12,4 | 601,3560 |  |  |  | C27H48N6O9 | 601,3556 | -0,8 | Desferrioxamine E | 1.14 |
|  | SL05 | 15,1 | 669,3821 |  |  |  | C31H52N6O10 | 669,3818 | -0,4 | New DFO congener | 1.14 |
|  | SL05 | 18,8 | 225,1485 |  |  |  | C13H20O3 | 225,1485 | 0,1 | Many isomers possible |  |
|  | SL05 | 21,7 | 912,6282 |  |  |  | C48H81N9O8 | 912,6281 | -0,2 | Surugamide A | 1.12 |
|  | SL05 | 23,5 | 511,2803 |  |  |  | C29H38N2O6 | 511,2803 | 0,0 | Polycyclic tetramate macrolactam | 1.2 |
|  | SL05 | 29,9 | 521,2497 |  |  |  | C26H36N2O9 | 521,2494 | -0,6 | Antimycin congener | 1.20 |
|  | SL05 | 31,2 | 535,2654 |  |  |  | C27H38N2O9 | 535,2650 | -0,7 | Antimycin congener | 1.20 |
|  | SL05 | 32,4 | 549,2811 |  |  |  | C28H40N2O9 | 549,2807 | -0,8 | Antimycin congener | 1.20 |
|  | SL01 | 9,5 | 561,3608 |  |  |  | C25H48N6O8 | 561,3606 | -0,3 | Desferrioxamine B | 2.16 |
|  | SL01 | 12,4 | 601,3554 |  |  |  | C27H48N6O9 | 601,3556 | 0,2 | Desferrioxamine E | 2.16 |
|  | SL01 | 18,8 | 716,2912 |  |  |  | C36H45NO14 | 716,2913 | 0,1 | Epelmycin C | 2.14 |
|  | SL01 | 19,8 | 841,3390 |  |  |  | C42H52N2O16 | 841,3390 | 0,0 | New cinerubin | 2.14 |
|  | SL01 | 22,2 | 826,3276 |  |  |  | C42H51NO16 | 826,3281 | 0,5 | Cinerubin B | 2.14 |
|  | SL01 | 25,4 | 392,2696 |  |  |  | C25H33N3O | 392,2696 | 0,0 | Propyl-metacyclooctylprodiginine | 2.5 |
|  | SL01 | 27,7 |  |  |  | 987,5481 |  |  |  | Potentially new NP |  |
|  | SL01 | 28,2 |  |  |  | 916,5110 |  |  |  | Potentially new NP |  |
|  | SL01 | 28,5 | 394,2855 |  |  |  | C25H35N3O | 394,2853 | -0,7 | Undecylprodigiosin | 2.5 |
|  | SL01 | 29,5 |  |  |  | 845,4739 |  |  |  | Potentially new NP |  |
|  | SL01 | 31,3 | 608,4524 |  |  |  | C35H61NO7 | 608,4521 | -0,6 | Pamamycin congener | 2.25 |
|  | SL01 | 32,1 | 622,4681 |  |  |  | C36H63NO7 | 622,4677 | -0,7 | Pamamycin congener | 2.25 |
|  | SL01 | 32,8 | 636,4837 |  |  |  | C37H65NO7 | 636,4834 | -0,5 | Pamamycin congener | 2.25 |
|  | SL02 | 13,2 | 254,2115 |  |  |  | C15H27NO2 | 254,2115 | -0,4 | Potentially new NP |  |
|  | SL02 | 16,3 |  |  |  | 313,1524 | C16H22N2O3 | 313,1523 | -0,5 | Inthomycin A or isomer | 2.3 |
|  | SL02 | 29,9 |  |  |  | 772,4602 | C41H67NO11 | 772,4606 | 0,6 | Venturicidin A | 2.4 |
